# Innovative nanostructured lipid-particles of apocynin and clove oil tagged with Chitin oligosaccharide for amelioration of tacrolimus-induced nephrotoxicity

**DOI:** 10.1038/s41598-025-13978-1

**Published:** 2025-08-08

**Authors:** Amir Elsayed Maghrabia, Mariza Fouad Boughdady, Sherry Mohamed Khater, Irhan Ibrahim Abu Hashim, Mahasen Mohammed Meshali

**Affiliations:** 1 Department of Pharmaceutics, Faculty of Pharmacy, Mansoura University, Mansoura, 35516 Egypt; 2 Department of Hospital Pharmacy, Urology and Nephrology Center, Mansoura University, Mansoura, 35516 Egypt; 3 Department of clinical pathology, Urology and Nephrology Center, Mansoura University, Mansoura, 35516 Egypt

**Keywords:** Apocynin, Clove oil, Nanostructured lipid carrier, Nephrotoxicity, Targeted, Phytopharmaceuticals, Drug delivery, Pharmaceutics, Kidney diseases

## Abstract

Tacrolimus (FK506) is a potent immunosuppressive agent widely employed to prevent allogeneic rejection in transplant recipients. However, its nephrotoxic effects pose significant limitations to long-term therapeutic use. To address this challenge, the present study aims to develop an innovative oral nano-delivery system designed to mitigate FK506-induced nephrotoxicity through the antioxidant properties of Apocynin (APO), clove oil (CO), and chitin oligosaccharide (CTOS). A nanostructured lipid carrier (NSLC) incorporating APO dissolved in CO and functionalized with CTOS was formulated using ultrasonic emulsification. Gelucire 43/01 and CO served as key lipid components in varying ratios. Physicochemical characterization of the developed NSLCs was conducted, assessing particle size (PS), polydispersity index (PDI), zeta potential (ZP), and encapsulation efficiency (EE%). The optimized formulation (F4) exhibited an EE% of 63.85 ± 1.98, PS of 123 ± 2.21 nm, PDI of 0.17 ± 0.09, and ZP of − 28 ± 1.98, demonstrating a biphasic release profile and stability under refrigerated conditions for six months. In vivo nephroprotective efficacy was evaluated in FK506-induced acute kidney injury (AKI) rat models, where oral administration of APO-loaded NSLCs significantly improved renal function and alleviated nephrotoxicity, as evidenced by biochemical markers and histopathological analyses. These findings underscore the potential of APO-loaded NSLCs as a promising oral phytopharmaceutical strategy for mitigating FK506-associated nephrotoxicity and enhancing therapeutic outcomes in transplant patients. Further studies are warranted to optimize and expand clinical applications.

**Supplementary Information:**

The online version contains supplementary material available at 10.1038/s41598-025-13978-1.

## Introduction

Nephrotoxicity is a rapid deterioration in kidney function that could progress to an advanced state of acute kidney injury (AKI) due to the toxic effects of medications or chemicals^1^. Nephrotoxic medications account for 6% of acute kidney injury (AKI) cases in the community and 20% of those acquired in hospital settings. In elderly patients, drug-induced nephrotoxicity is responsible for as much as 66% of AKI cases that develop during hospitalization^2^. Reactive oxygen species (ROS) are significant contributors to the pathogenesis of drug-induced nephrotoxicity, primarily due to the urinary system’s heightened vulnerability to oxidative stress. Furthermore, ROS are considered pivotal in reducing glomerular filtration rate (GFR) and tubular necrosis^3^.

Tacrolimus, commonly known as FK506, is a lipophilic macrolide that has been isolated from the bacterium *Streptomyces tsukubaensis*. It is classified as a calcineurin inhibitor (CNI) and functions as an important immunosuppressant. FK506 plays a crucial role in immunosuppressive therapy, especially in organ transplantation, as it significantly lowers the likelihood of graft rejection^2^. The kidneys are particularly vulnerable to the toxic effects of FK506, a phenomenon attributed to its broad distribution throughout the body. The mechanism underlying FK506-induced nephrotoxicity is thought to involve the production of reactive oxygen species (ROS) via the activation of the NADPH oxidase (NOX) pathway^4^.

Phytomedicines are plant-derived active compounds associated with remedial and restorative properties^5^. Fortunately, there are many bioactive antioxidant phytochemicals isolated from medicinal plants that have been proven to diminish oxidative stress^6^. They possess strong antioxidant and free radical scavenging abilities, as well as anti-inflammatory action^7^. Apocynin (4-hydroxy-3-methoxy-acetophenone), a naturally occurring methoxy-substituted catechol, is an inhibitor of NADPH oxidases (NOXs). It is extracted from the roots of Apocynum cannabinum (Canadian hemp) and Picrorhiza kurroa (Scrophulariaceae)^8^. It is soluble in hot water (2 mg/ml) and certain organic solvents like ethanol, DMSO, and DMF. It is poorly soluble in cold water (pKa: 8.17)^9^. It demonstrates diverse pharmacological features, encompassing antiasthmatic, antioxidant, antiallergic, anticancer, and hepatoprotective^10,11^. The inhibition of NOX has indicated potential therapeutic effects in numerous diseases such as arteriosclerosis, arthritis, diabetic nephropathy, and nephrotoxicity induced by cyclosporine^12–15^. Additionally, APO has exhibited inhibitory effects on pro-inflammatory cytokines and apoptosis. It also has a perfect safety profile in long-term animal studies^3^. Notwithstanding the extensive range of activities exhibited by APO, its clinical efficacy is constrained due to pharmacological impediments, including inadequate aqueous solubility, suboptimal oral bioavailability (10%), and rapid metabolic clearance^3^.

Clove oil (CO), an essential oil obtained from the clove buds of *Syzygium aromaticum*, demonstrates considerable antioxidant, antibacterial, antifungal, anesthetic, and analgesic activity^16–18^. Its primary constituent is eugenol, a phenylpropanoid that typically comprises 50–90% of the oil depending on the extraction method and plant origin. Other notable components include eugenol acetate, β-caryophyllene, and α-humulene, which contribute to its aromatic profile and pharmacological properties. These compounds exhibit potent antioxidant, anti-inflammatory, antimicrobial, and analgesic activities, making clove oil a valuable agent in pharmaceutical applications^19^. Eugenol, the predominant phenolic constituent of CO, accounts for its therapeutic effects^20^. It profoundly inhibits the metabolic performance of enzymes participating in FK506 metabolism in a dose-dependent manner^21^. CO is prone to chemical instability when subjected to air, moisture, light, and temperature changes^22^. To address these issues, the integration of CO into a nanoscale system is a viable approach to enhance its stability, solubility, and effectiveness^23^.

CTOS are oligomers composed primarily of β−1,4-linked N-acetyl-D-glucosamine (GlcNAc) units, with occasional D-glucosamine (GlcN) residues. They are derived from the partial hydrolysis of chitin. CTOS typically have a polymerization degree of 2–6, which confers water solubility, pH stability, and bioactivity. Their molecular weight ranges from 0.2 to 3.0 kDa, depending on the hydrolysis method (enzymatic or chemical)^24^. It is notable for its solubility in water, thermal stability, biodegradability, and non-toxicity, along with its ability to undergo chemical modifications. Beyond its function as a drug delivery system, CTOS is associated with numerous biological effects, including anti-cancer, anti-inflammatory, and anti-microbial activities. Furthermore, it is regarded as a potential inhibitor of P-glycoprotein^25^. Upon degradation, CTOS is primarily distributed and eliminated through the kidneys, with around 80% being excreted in urine. Notably, CTOS has been shown to possess reno-protective properties in cases of drug-induced nephrotoxicity and renal dysfunction, highlighting its critical involvement in renal pathologies^26^.

Employing nanoparticles in drug delivery systems can significantly increase drug bioavailability while enabling precise targeting^27^. Nanostructured Lipid Carriers (NSLCs) are novel lipid-based nanoparticles formed by mixing spatially different lipids (solid and liquid). They have emerged as a highly effective vehicle for enhancing oral bioavailability in various applications^28,29^. NSLCs measuring between 1 and 160 nm in diameter can engage with different regions of the kidney through several mechanisms^30^. NSLCs established with essential oils enable protection against uncontrolled volatilization, oxidation, and environment-triggered degradation, besides providing controlled drug release^31^. They are easy to prepare, industrially scalable, and offer extended release of drugs because of the solid lipid-liquid lipid matrix. To the best of our knowledge, there is no issued information yet concerning the assessment of APO, CO, and CTOS effects on nephrotoxicity induced by FK 506, despite being promising natural bioactive phytopharmaceuticals. From here, the objective of the present study was to explore the feasibility of formulating APO, CO, and CTOS into the NSLC system to improve their oral bioavailability and renal delivery. This novel NSLCs of APO, by its NOX inhibition, besides the antioxidant eugenol content and CTOS’ reno-protective effect, might attenuate FK 506-induced renal damage.

## Materials and methods

### Materials

APO, Tween 80 (CAS NO. 9005-65-6), and Carboxymethylcellulose (CMC) sodium powder (cas.no: 9004–32 − 4) were sourced from Sigma-Aldrich (Saint Louis, USA). CO was attained from FUJIFILM WAKO, USA. CTOS was kindly provided by Yaizu Suisankagaku (Shizuoka, Japan). Gattefosse (St Priest, France) graciously sent GE 43/01 (CAS NO. 85665-33-4, HLB: 1, Melting point: 43 °C) as a gift. El-Nasr Pharmaceutical Chemical Co. (Cairo, Egypt) provided an analytical grade of hydrochloric acid 33% (HCl), disodium hydrogen phosphate (Na_2_HPO4), and monobasic potassium phosphate (KH_2_PO4). The creatinine reagent kit (Beckman Synchron LX Systems BK-472525D), along with kits for blood urea nitrogen (BUN), albumin (ALB), and total proteins, were all obtained from Diamond Diagnostics Inc., USA. Serum neutrophil gelatinase-associated lipocalin (NGAL), kidney injury molecules (KIM-1), and cystatin C ELISA kits were acquired from MyBioSource, USA.

### Methods

Preliminary saturation solubility studies were conducted to find suitable lipids, surfactants, and optimum sonication parameters for the preparation of NSLCs. Then, the binary mixture ratio of both the solid lipids and liquid lipids was selected based on the differential scanning calorimeter (DSC) studies. Fourier transform infrared (FTIR) studies and thermal analysis were conducted to interpret the compatibility between the lipids and the drug^32–35^.

#### Preparation of APO-loaded NSLCs

The APO-loaded NSLCs were prepared through emulsification followed by ultrasonication^36^. The selection of both solid and liquid lipids was based on the high solubilization extent of the drug^37^. Gelucire 43/01 is a lipid-based excipient composed predominantly of triglyceride esters of medium- to long-chain fatty acids (C8–C18), along with mono- and diglycerides. Its low melting point (~ 43 °C) renders it particularly suitable for the encapsulation and protection of active pharmaceutical ingredients (APIs), especially those sensitive to thermal or oxidative degradation. The simplicity and efficiency of API inclusion within Gelucire 43/01 make it a valuable carrier in lipid-based formulations, especially for enhancing the stability and performance of sensitive therapeutic agents^38^. In this investigation, Gelucire 43/01 was chosen as a solid lipid because of its exceptional hydrophobicity (HLB: 1), low density, and melting point that exceeds that of the human body, whereas CO acts as the liquid lipid^23,39^. A mixture of CTOS with surfactant (Tween 80) served as an external aqueous phase. The water-soluble CTOS provides antimicrobial properties for the external aqueous phase^40^. Tween 80, chemically known as polyoxyethylene (20) sorbitan monooleate, is a nonionic surfactant widely employed in pharmaceutical formulations for its solubilizing and stabilizing properties. Structurally, it consists of a sorbitan backbone esterified with oleic acid and conjugated to approximately 20 ethylene oxide units, imparting significant hydrophilicity and amphiphilic behavior. It belongs to the polysorbate class of amphiphiles. It enhances the aqueous solubility of hydrophobic APIs. It has a good emulsification ability for lipid mixtures (HLB ~ 15 ideal for o/w emulsion), with the formation of stable NSLCs^41,42^.

The Lipid phase (1 g) consisted of solid lipid (Gelucire^®^ 43/01) and liquid lipid (CO) in different ratios. In contrast, the aqueous phase consisted of 0.5% (w/w) CTOS and 1% v/v of the hydrophilic emulsifier (Tween^®^ 80) dissolved in 20 mL of distilled water, then filtered by 0.45 μm membrane filters (EMD Millipore, Billerica, MA, USA). The amounts of solid and liquid lipids in each formula are presented in Table 1. GE 43/01 was heated separately to 5 °C above the solid lipid transition temperature. APO was dissolved in the molten lipid phase and subsequently mixed with CO. The molten lipid phase was then added dropwise to the aqueous phase, which had been heated to align with the temperature of the lipid phase. The resulting mixture was stirred at 1000 rpm for 5 min to form a pre-emulsion, facilitated by a magnetic stirrer (Magnetic stirrers, Thermolyne Corporation, Dubuque, Iowa, USA). The produced pre-emulsion was then sonicated for 5 min at the following settings (Amplitude: 90%, Timer: 5 min, Pulser: 1 s ON/1 s OFF, probe temperature: room temperature (25 ± 1 °C)), using a probe sonicator (Sonics Vibra-cell™, Model VC 505, Sonic & Materials, Inc., USA, Probe model CV 334, Serial No. 2013020605), to form the NSLCs. APO-loaded NSLCs were isolated by centrifugation at 13,000 rpm for 30 min at −4 °C (Cooling centrifuge, CE16-4 × 100RD, ACCULAB, USA), followed by washing with deionized water and freeze-drying under vacuum at −80 °C (Freeze dryer, SIM FD8-8T, SIM International, USA). The lyophilized NSLCs were stored at 4 °C to be further subjected to characterization studies. The supernatant would be saved for indirect determination of EE%. Plain NSLCs corresponding to each formula were prepared using the same ingredients, except for APO to be used as a blank.

**Table 1:** Composition of APO-loaded NSLCs.

Formula	GE 43/01 weight (gm)	CO weight (gm)
F1	1	
F2	0.9	0.1
F3	0.8	0.2
F4	0.7	0.3
F5	0.6	0.4
F6	0.5	0.5

200 mg of APO, 0.5% w/v CTOS, and 1% of Tween 80 were constant in all formulae.

#### Characterization and optimization of APO-loaded NSLCs

##### Encapsulation efficiency (EE%)

The estimation of the encapsulation efficiency (EE%) for APO-loaded NSLCs was performed indirectly by measuring the free APO concentrations in the clear supernatants collected after centrifugation at 13,000 rpm for 30 min^13^. Subsequently, 0.1 mL of the clear supernatant was diluted to a final volume of 100 mL using deionized water and analyzed with a spectrophotometer (UV/VIS Spectro, double beam, Labomed Inc., USA). To mitigate potential interference from CO, the estimation of APO (EE%) was conducted at 307 nm, utilizing the supernatant of plain NSLCs as a blank reference^8^:

$$\:\:\varvec{E}\varvec{E}\:\varvec{\%}\:=\frac{\varvec{t}\varvec{o}\varvec{t}\varvec{a}\varvec{l}\:\varvec{a}\varvec{m}\varvec{o}\varvec{u}\varvec{n}\varvec{t}\:\varvec{o}\varvec{f}\:\varvec{t}\varvec{h}\varvec{e}\:\varvec{d}\varvec{r}\varvec{u}\varvec{g}-\varvec{a}\varvec{m}\varvec{o}\varvec{u}\varvec{n}\varvec{t}\:\varvec{o}\varvec{f}\:\varvec{u}\varvec{n}\:\varvec{e}\varvec{n}\varvec{t}\varvec{r}\varvec{a}\varvec{p}\varvec{p}\varvec{e}\varvec{d}\:\varvec{d}\varvec{r}\varvec{u}\varvec{g}\:\varvec{i}\varvec{n}\:\varvec{t}\varvec{h}\varvec{e}\:\varvec{s}\varvec{u}\varvec{p}\varvec{e}\varvec{r}\varvec{n}\varvec{a}\varvec{n}\varvec{t}\varvec{a}\varvec{n}\varvec{t}}{\varvec{t}\varvec{o}\varvec{t}\varvec{a}\varvec{l}\:\varvec{a}\varvec{m}\varvec{o}\varvec{u}\varvec{n}\varvec{t}\:\varvec{o}\varvec{f}\:\varvec{t}\varvec{h}\varvec{e}\:\varvec{d}\varvec{r}\varvec{u}\varvec{g}\:}\varvec{x}100\:\:$$

##### Particle size (PS) and polydispersity index (PDI)

PS and PDI of the freshly prepared NSLCs were determined using the Malvern Zetasizer Nano series (Malvern Instruments Limited, UK) after proper dilution (0.1 mL of the formed dispersion was diluted to 10 mL with deionized water)^43^.

##### Zeta potential (ZP)

Zeta potential (ZP) measurements for the freshly prepared APO-loaded NSLC samples were conducted after appropriate dilution with deionized water. In this process, 0.1 mL of the dispersion was diluted to 10 mL with deionized water. The analysis was performed using the Malvern Zetasizer Nano series (Malvern Instruments Limited, UK) through the Laser Doppler Anemometry (LDA) method^43^.

#### Optimization of APO-loaded NSLCs

Optimization of the prepared APO-loaded NSLCs was based on acquiring stable NSLCs with acceptable ZP value, and maximum EE% % while keeping PS within the acceptable range for targeted renal delivery (< 200 nm)^27^.

##### Evaluation of the optimal APO-loaded NSLCs (F4)

######  Fourier transform infrared spectroscopy (FT-IR)

An FT-IR spectrophotometer (Madison Instruments, Middleton, Wisconsin, USA) was employed for the chemical characterization of APO, CO, GE, CTOS, their physical mixture (aligned with the optimal formula), the plain optimal formula, and the optimal formulation (F4). Potassium bromide discs were created using a hydrostatic press. The scanning range was set between 4000 and 500 cm^−1^. Each sample was ground, combined with potassium bromide, and compressed before measurement^8^.

###### Differential scanning calorimetry (DSC)

Differential Scanning Calorimetry (DSC) was employed to evaluate APO, CO, GE, CTOS, their physical mixture (aligned with the optimal formula), the plain optimal formula, and the optimal formula (F4). The analysis was conducted using a Perkin-Elmer Differential Scanning Calorimeter (Perkin-Elmer 4, USA), which has been calibrated with indium of 99.99% purity, exhibiting a melting point of 156.6 °C. For the experiment, eight milligrams of each sample were encapsulated in standard aluminum pans and heated from 30 to 300 °C at a rate of 10 °C/min, with a continuous flow of dry nitrogen at 20 mL/min. An empty pan, sealed similarly to the samples, was utilized as a reference^8^.

######  X-ray diffractometry (XRD)

The X-ray diffractograms for APO, CO, GE, CTOS, their physical mixture (aligned with the optimal formula), the plain optimal formula, and the optimal formulation (F4) were obtained through a Diano X-ray diffractometer (USA) utilizing Co-Kα radiation (45 kV, 9 mA), scanning from 3 to 50° at a 2θ angle^8^.

###### Transmission electron microscopy (TEM)

The morphological analysis of the optimal formulation (F4) was conducted utilizing a JEOL TEM (100 CX, Japan). One milliliter aliquot of the freshly prepared lipid dispersion (F4) was diluted tenfold with ultrapure water and subjected to sonication in an ultrasonic bath for 5 min. A single drop of the resulting diluted sample was spread onto a Formvar-coated copper grid (200 mesh, Science Services, Munich, Germany). Excess material was removed using filter paper, resulting in a thin film that spanned the grid’s apertures. Following complete evaporation at ambient temperature, the samples were analyzed and imaged using digital microscopy and soft imaging techniques^22^.

###### Scanning electron microscopy (SEM)

The surface morphology of the optimal lyophilized APO-loaded NSLCs (F4) was assessed utilizing a JEOL scanning electron microscope (JSM-6510LV, Japan). The samples were affixed to aluminum stubs using conductive double-sided adhesive tape and subsequently coated with gold via a sputter coater at a current of 50 milliamperes (mA) for 50 s^44^.

###### In vitro release study

Modified vertical Franz diffusion cells were utilized to examine the diffusion characteristics of APO derived from the optimal formulation (F4) and a control aqueous solution (2 mg/mL) of the free drug. In vitro release studies were performed in three different dissolution media: 0.1 N HCl at pH 1.2, phosphate buffer at pH 6.8, and phosphate buffer at pH 7.4, which represent the pH environments of the stomach, intestine, and blood, respectively. The Franz diffusion cells, each with a diameter of 3 cm, were placed in a shaking incubator (GFL Gesellschaft für Labortechnik, Burgwedel, Germany) and maintained at a temperature of 37 ± 0.5 °C throughout the experiment.

A Spectra/Por™ cellulose membrane (with a molecular weight cut-off of 12,000–14,000 Da, Spectrum Medical Industries Inc., Los Angeles, USA) was equilibrated with the release medium for 12 h before being installed in the diffusion cell. The membrane was firmly positioned between the donor and receptor compartments. A total of ten milligrams of the optimal formulation (F4), corresponding to 2 mg of APO, was placed in the donor compartment. The receptor compartment was filled with 100 mL of dialysis medium and stirred at 100 rpm. At specified time intervals, 1 mL aliquots of the release medium were taken from the receptor compartment and replaced with an equal volume of fresh medium to ensure sink conditions were maintained throughout the experiment. The collected samples were filtered through a Millipore filter (0.45 μm, Berlin, Germany) and analyzed for drug concentration using a UV–VIS spectrophotometer at 307 nm following appropriate dilution. Each experiment was conducted in triplicate, and the cumulative percentage of APO released was calculated at each time point. Simultaneously, an aqueous solution containing the same quantity of APO was assessed for diffusion in a comparable manner, also in triplicate^45^.

####### Kinetic analysis of the drug release data

Various kinetic models, including zero-order, first-order, and Higuchi’s model, were employed to fit the release data^46^. In addition, the first 60% of the release data were analyzed using the Korsmeyer–Peppas kinetic model to ascertain the release mechanism, represented by the equation^47^:

$$\:\raisebox{1ex}{${\varvec{M}}_{\varvec{t}}$}\!\left/\:\!\raisebox{-1ex}{${\varvec{M}}_{\varvec{\infty\:}}$}\right.=\varvec{K}{\varvec{t}}^{\varvec{n}}$$

where M_t_/M_∞_ is the fraction of drug released after time t, n is the characteristic diffusional exponent, and k is the release rate constant.

The Weibull model, which serves as an empirical representation of the release pattern for the optimal formulation (F4), was also applied to the release data, as indicated by the Eq^8^.:

$$\:\varvec{ln}[-\varvec{ln}(1-\varvec{F})]=\varvec{\beta\:}\varvec{ln}\varvec{t}\varvec{d}+\varvec{\beta\:}\varvec{ln}\varvec{t}$$

In this equation, F represents the fraction of drug released at time t, β is a shape parameter that defines the release curve, and td is a location parameter indicating the lag time before drug release commences. The selection of the kinetic model that best fits the release profile was based on the highest coefficient of determination (R²) and the lowest Akaike information criterion (AIC) values^8^.

###### Stability study

A stability study under accelerated conditions was conducted to evaluate the stability of APO-loaded NSLCs. Freshly prepared lyophilized optimal formula (F4) samples were placed in securely sealed glass containers and stored under two distinct conditions: refrigerated (4 ± 1 °C) and at an ambient temperature (25 °C ± 2 °C/60% RH ± 5% RH) for six months. The stability of the chosen formulation was analyzed by measuring particle size (PS), polydispersity index (PDI), zeta potential (ZP), and encapsulation efficiency percentage (EE%) at the initial time point and subsequently every month throughout the six-month storage period^18^.

###### In vivo assessment of the optimal APO-loaded NSLCs (F4) effect against FK506-induced AKI in rats

####### Animals

In this study, male Sprague-Dawley rats, each weighing between 180 and 200 g, were acclimatized for one week before experimentation. The animals were housed under optimal temperature conditions of 20 to 25 °C, with a regulated 12-hour light/dark cycle. They were given free access to water and a standard laboratory diet ad libitum in the animal house of the Medical Experimental Research Center (MERC) at Mansoura University. All experimental protocols were conducted in compliance with the “Principles of Laboratory Animal Care” as per the National Institute of Health Publication No. 85 − 23 (updated 1985) and were approved by the Ethical Committee of the Faculty of Pharmacy at Mansoura University, Egypt (Ethical Approval Code: 2024 − 163). Additionally, the research adhered to the ARRIVE guidelines for the reporting of in vivo studies.

####### Experimental protocol

To evaluate the pharmacodynamic effects of the optimal formulation (F4) on FK506-induced nephrotoxicity in rats, a total of twenty-four rats were systematically allocated into four groups, each consisting of six rats.:

- **Group I**: (normal control), rats received only 2 mL of 0.5% (w/v) carboxy methyl cellulose (CMC) P.O.
- **Group II**: (positive control), nephrotoxicity was induced in rats by administration of a total dose of 3 mg FK506 suspended in 2 mL 0.5% (w/v) CMC P.O.
- **Group III**: (Free APO group), rats received 2mL of (15 mg pure APO + 3 mg FK506) suspension in 0.5% (w/v) CMC) P.O.
- **Group IV**: (APO-loaded NSLCs group), rats received 2mL (APO-loaded NSLCs (F4) equivalent to 15 mg pure APO + 3 mg FK506) suspension in 0.5% (w/v) CMC) P.O.

In this experimental design, all groups underwent daily oral pretreatment via gastric gavage for seven days. Upon completion of the experiment, blood samples were obtained from the tail vein, and serum was separated through centrifugation at 3000 g for 10 min using a Hettich Micro 22R centrifuge (Germany). The serum was subsequently frozen at − 80 °C for the analysis of renal function parameters. On the eighth day, all rats were euthanized through the administration of ketamine HCL (80 mg/kg, intraperitoneally) and xylazine HCL (10 mg/kg, intraperitoneally)^48^. The kidneys were then excised, rinsed in ice-cold physiological saline, and prepared for histopathological evaluation^13^.

####### Effect of APO on FK506-induced changes in body and kidney weights

The experimental rats were subjected to weighing both at the outset of the experiment and again before scarification, employing the OHAUS weight measurement scale (OHAUS CORPORATION, USA). Furthermore, the isolated kidneys were meticulously cleaned of all extraneous tissue before weighing^49^.

####### Effect of APO on FK506-induced changes in kidney function in rats

The assessment of serum creatinine (Scr), blood urea nitrogen (BUN), and total proteins was conducted using an auto-analyzer (ILab-300-Biomerieux Diagnostic, Milano, Italy). Furthermore, serum concentrations of neutrophil gelatinase-associated lipocalin (NGAL), kidney injury molecule-1 (KIM-1), and cystatin C were evaluated through ELISA kit methodologies, adhering to the instructions outlined by the kit manufacturer (MyBioSource, USA)^50^.

####### Histopathological analysis

At the end of the experiment, the isolated kidneys were quickly fixed in 10% buffered formalin, then embedded in paraffin and sectioned into 5-mm thick slices, which were stained with hematoxylin and eosin (H&E). The slides were analyzed under a Nikon Eclipse Ci microscope connected to a Kameram^®^ Digital Image Analysis System. The pathologist performing the histopathological assessment was blinded to the experimental design^3^.

##### Statistical analysis

The experimental data obtained from both in vitro and in vivo studies were expressed as mean ± standard deviation (SD) for *n* = 3 and mean ± standard error of the mean (SEM) for *n* = 6. Statistical analyses were conducted utilizing GraphPad Prism Software Inc. (San Diego, CA, version 9.3.1). For parametric data, a one-way analysis of variance (ANOVA) was performed, followed by the Tukey-Kramer test to facilitate multiple comparisons. A *p*-value threshold of less than 0.05 was established to determine statistical significance.

## Results & discussion

### Characterization of APO-loaded NSLCs

#### Encapsulation efficiency (%EE)

As shown in Table 2, all prepared formulae have a high % EE with values ranging from 47.71 ± 2.24% (F1) to 63.85 ± 1.98% (F4). These results demonstrate the ability of the prepared NSLCs to encapsulate APO. However, a closer examination of the results depicts that a reduction in the solid-to-liquid lipid ratio in different formulae led to a noteworthy increment in %EE (F1:F4). This could be justified as increasing the amount of liquid lipid (CO) would help in increasing APO solubility in the lipid mixture by providing more space for drug incorporation (crystal imperfections)^51^. This effect is up to a distinct limit after which the viscosity of the lipid mixture decreases, allowing the drug to escape with a consequent reduction of EE%, as perceived in the EE% of F5 and F6 were 59.29% and 52.46% respectively^52^.

**Table 2:** Characterization of the prepared APO-loaded NSLCs.

Formula	Encapsulation efficiency (%)	Particle size (nm)	Polydispersity index (PDI)	Zeta potential (mV)
F1	47.71 ± 2.24%	223 ± 5.46 nm	0.34 ± 0.16	−40 ± 2.30
F2	51.21 ± 1.53%	167 ± 4.16 nm	0.29 ± 0.12	− 36 ± 1.90
F3	56.22 ± 2.14%	149 ± 3.45 nm	0.21 ± 0.10	−31 ± 2.10
F4	63.85 ± 1.98%	123 ± 2.21 nm	0.17 ± 0.09	−28 ± 1.98
F5	59.29 ± 1.33%	119 ± 3.44 nm	0.18 ± 0.09	−27 ± 1.55
F6	52.46 ± 1.21%	117 ± 3. 56 nm	0.19 ± 0.10	−27 ± 1.23

Each value represents the mean ± SD (*n* = 3).

See Table 1 for formula composition.

#### Particle size (PS) and polydispersity index (PDI)

PS is the most influential factor in determining the different locations of nanomaterial accumulation in the kidneys^53^. This may be attributed to the size selectivity of the glomerular filtration barrier (GFB)^27^. It has a profound impact on the rate and extent of drug release from the system. Repaglinide-loaded nanostructured lipid carriers with different particle sizes for improving oral absorption^54^. As depicted in Table 2, the PS and PDI of the developed APO-loaded NSLCs vary between 117 ± 3.56 (F6) to 223 ± 5.46 nm (F1), and 0.17 ± 0.09 (F4) to 0.34 ± 0.16 (F1), respectively. Both characteristics (PS and PDI) exhibited a pronounced decrease in response to a lower solid lipid (GE) to liquid lipid (CO) ratio, as evidenced in F1:F4. A possible justification for this phenomenon could lie in the solidification process during NSLC preparation, where a higher concentration of solid lipids is prone to fusing or aggregating. These aggregates may remain intact, resulting in the emergence of larger particles with a broader size distribution^55^.

#### Zeta potential (ZP)

In the context of formulation stability, ZP is particularly important, as it provides insights into the surface charge characteristics of the particles. For a stable NSLC system, an absolute value of 30 mV is essential, where the electrostatic repulsion between the particles could keep them away and separate from each other^56^. As outlined in Table 2, APO-loaded NSLCs showed electronegative ZP values between − 27 ± 1.23 mV (**F1**) and − 40 ± 2.3 mV **(F6)**. Such pronounced high negative ZP signifies powerful repulsive forces among the particles, which successfully deter the aggregation of internal phases^57^. Encouragingly, other investigations have reported analogous ZP values in studies that used GE 43/01 as the solid lipid for the creation of NSLCs^57^. The anionic nature of the solid lipid GE 43/01 matrix in the maturation medium is responsible for conferring such a negative charge^58,59^. Moreover, the hydroxyl groups associated with the amphiphilic surfactant Tween 80 are expected to be adsorbed at the interface, contributing to the formation of an electronegative charge^58^. In contrast to chitosan, known for imparting a positive charge to nanoparticles, CTOS did not alter the electronegative charge of the formulated NSLCs^45^. Rather, a notable increase in the electronegative ZP value was recorded, especially as the amount of solid lipid (GE 43/01) increased. The surface charge of NSLCs plays a crucial role in influencing their renal filtration characteristics as they control their accumulation or clearance by the kidney^60^. The negative charges of heparan sulfate in the glomerular basement membrane (GBM) and glomerular filtration barrier (GFB) form a charge-selective barrier. Positively charged nanomaterials exhibit a higher propensity to traverse the glomerular filtration barrier (GFB) and the renal tubular system, resulting in their elimination by the kidneys^53^. Consequently, the developed negatively charged APO-NSLCs represent a promising approach to extending their retention duration within the renal system^53^.

### Optimization of APO-loaded NSLCs

In alignment with the predefined optimization criteria, **F4** (0.7 gm GE and 0.3 gm CO, Table 2) was identified as the optimal formula and subjected to subsequent assessment.

#### Evaluation of the optimal APO-loaded NSLCs (F4)

##### Fourier transform infrared spectroscopy (FT-IR)

**Fig. 1:**
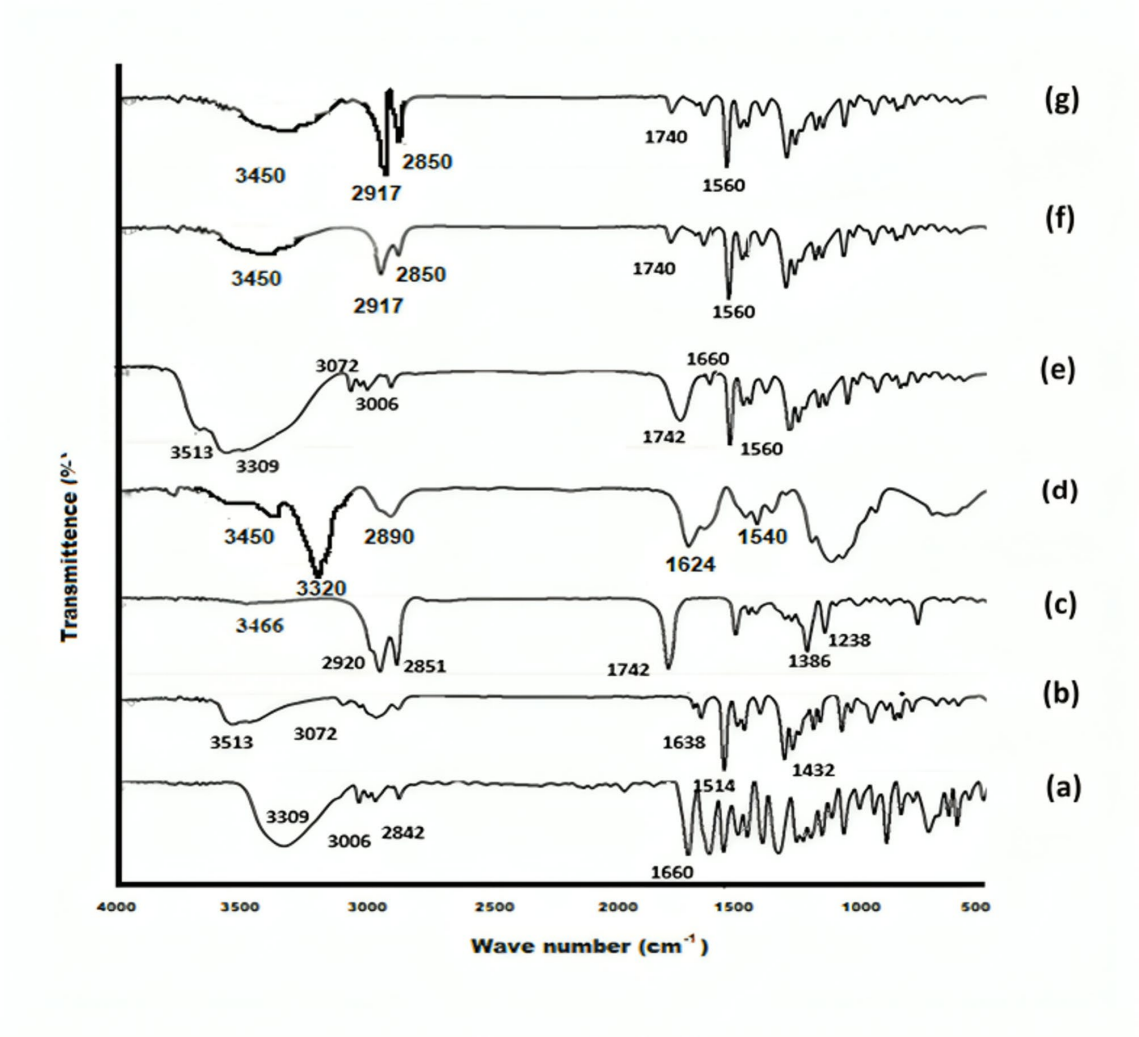
FT-IR spectra of (**a**) APO, (**b**) CO, (**c**) GE, (**d**) CTOS, (**e**) physical mixture, (**f**) plain optimal formula, and (**g**) optimal formula (**F4**).

The FT-IR spectra of the optimal formulation (F4) alongside its components are presented in Fig. 1. As denoted in (Fig. 1a), the functional conjugated ketone bonds (C = O), alkane carbon-hydrogen, aromatic hydrogen, and phenolic OH of APO were represented by various infrared shoulders respectively at 1660, 2842, 3006, and 3309 cm^−1^^45^. The FT-IR spectrum of CO, illustrated in Fig. 1b, revealed a significant peak at 3072 cm^−1^, which is associated with O–H stretching. Additionally, the spectrum indicated peaks for eugenol at 1638 and 1514 cm^−1^, while the peak at 1432 cm^−1^ was attributed to C–C stretching vibrations of the phenyl ring^61^. The prominent peaks associated with GE (43/01) were identified at 3466 cm^−1^ (OH stretching), 2920 and 2851 cm^−1^ (C-H stretching), 1742 cm^−1^ (C = C stretching), and between 1238 and 1386 cm^−1^ (C-O-C stretching), as shown in Fig. 1c^62^. On the other hand, the spectra of CTOS display a range of narrow absorption bands, typical of crystalline polysaccharide samples. The region corresponding to the C = O stretching of the amide moiety displays two distinct peaks at 1624 cm^−1^ and 1540 cm^−1^, which are characteristic of secondary and primary amides, respectively (Fig. 1d). The lack of splitting in the primary amide band indicates that CTOS is present in the β-form^63^. The stretching vibrations of N-H and O-H and intramolecular hydrogen bonding manifest as two prominent bands at 3320 and 3450 cm^−1^. Previous studies have documented similar bands for CTOS^64^. In the spectrum of the physical mixture (Fig. 1e), individual ingredient bands are identifiable; however, some bands were either reduced in intensity or completely absent due to the dilution effect. The plain optimal formula and its corresponding optimal formulation (F4) exhibited characteristic peaks of CTOS at 3450 and 1560 cm^−1^, while the peak at 1740 cm^−1^ was linked to GE. Moreover, the figure prints of CO disappeared from the spectrum of the optimal and the corresponding plain one, indicating the successful formation of NSLCs. In contrast, the disappearance of APO peaks from the spectrum of the optimal formula (F4) coincides with drug incorporation in the lipid core of the formed NSLCs (Fig. 1f **& g**).

##### Differential scanning calorimetry (DSC)

**Fig. 2:**
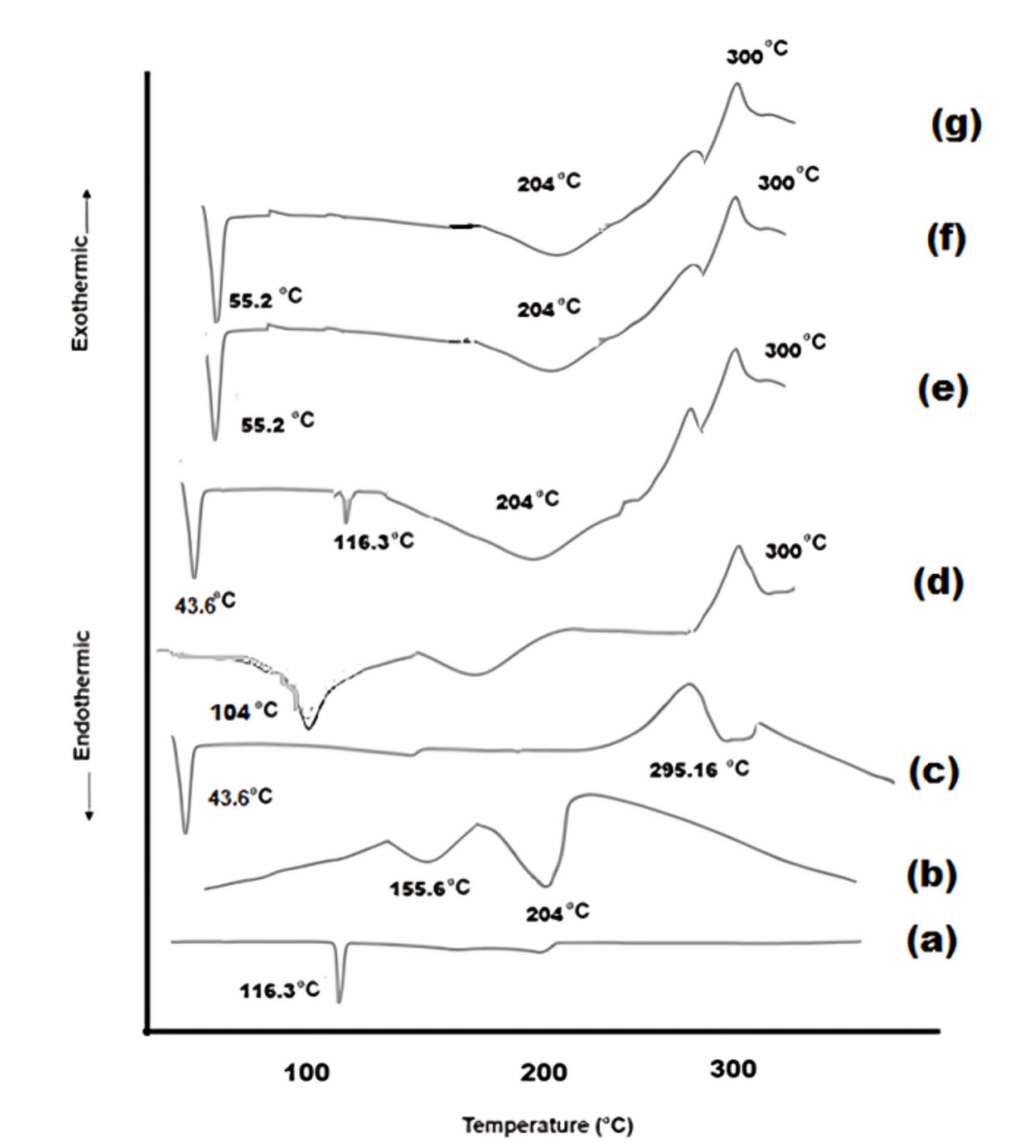
DSC spectra of (**a**) APO, (**b**) CO, (**c**) GE, (**d**) CTOS, (**e**) physical mixture, (**f**) plain optimal formula, and (**g**) optimal formula (F4).

Differential Scanning Calorimetry (DSC) is instrumental in identifying polymorphic transitions within formulations, thereby elucidating their thermodynamic characteristics and potential interactions with drugs^41^. The DSC profiles for the optimal formulation (F4) and its components are presented in Fig. 2. APO demonstrated a melting endotherm at 116.3 °C (Fig. 2a), signifying its crystalline structure^45^. Pure CO exhibited distinct endothermic reactions at 155.6 °C and 204 °C, which are likely attributable to boiling and evaporation processes **(**Fig. 2b**)**^43^. The DSC trace for GE 43/01 (Fig. 2c) revealed an endothermic peak at 43.6 °C, corresponding to the melting of GE, and an exothermic peak at 295.16 °C^65^. CTOS (Fig. 2d) displayed a broad endothermic peak at 104 °C, linked to the evaporation of water in the sample due to polymer dehydration and breakdown, and an exothermic peak around 300 °C, indicative of polymer decomposition^66–68^. The DSC thermogram of the physical mixture showed all characteristic peaks of the individual components at their respective positions, with a noticeable reduction in the APO peak due to the diluting effect (Fig. 2e). The DSC of both the plain optimal formula and the optimal formula (F4) exhibited a slight shift in the GE peak and a reduction in the endothermic peaks of CO and CTOS (Fig. 2f **& g**), confirming the successful formation of NSLCs. The significant disappearance of the APO peak in the F4 spectrum (Fig. 2g) indicates drug encapsulation within the NSLCs. These results, supported by FT-IR analysis, confirm the incorporation of APO within the NSLC system.

##### X-ray diffractometry (XRD)

**Fig. 3:**
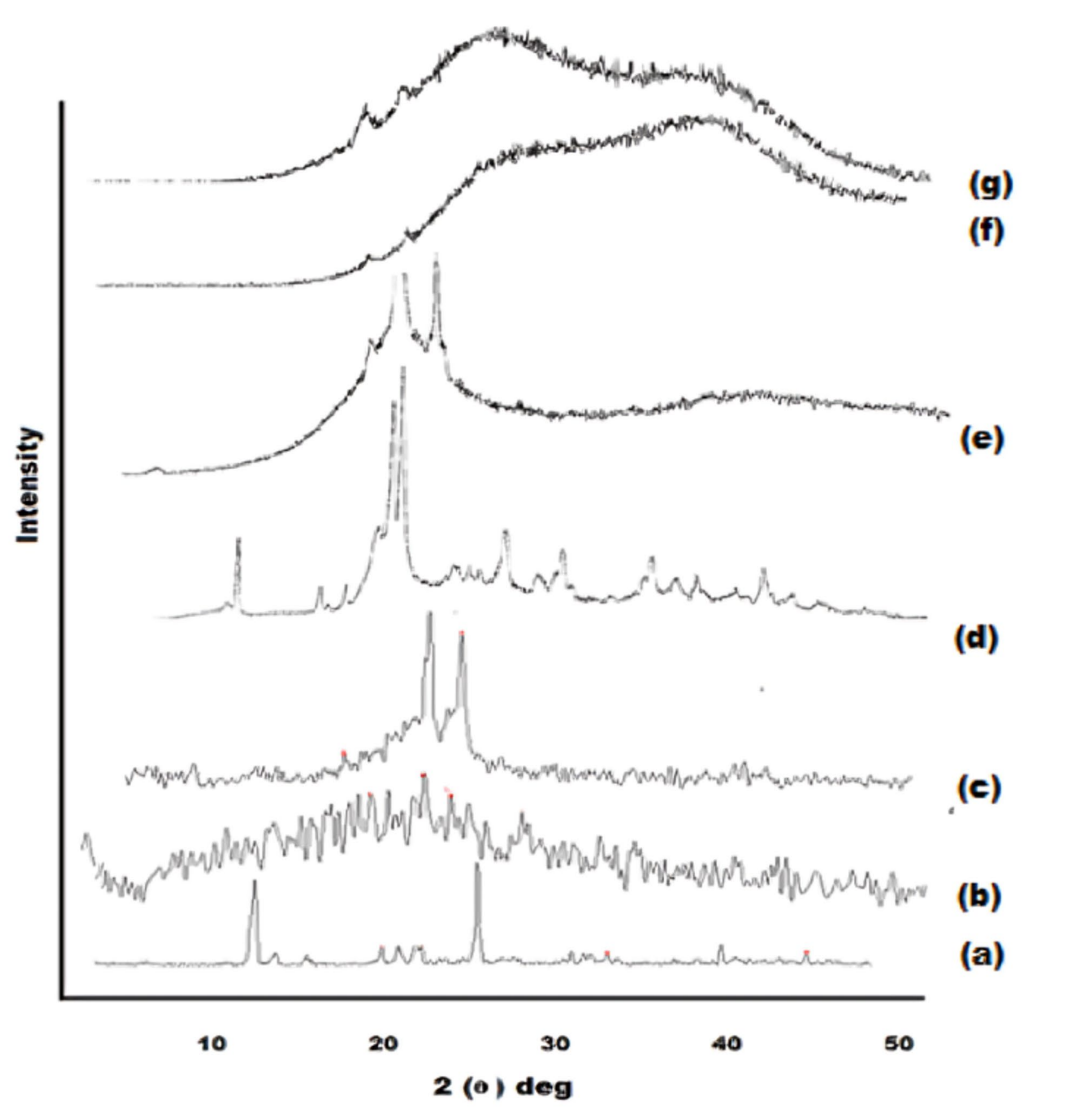
XRD diffractograms of (**a**) APO, (**b**) CO, (**c**) GE, (**d**) CTOS, (**e**) physical mixture, (**f**) plain optimal formula, and (**g**) optimal formula (F4).

The structural characteristics, whether crystalline or amorphous, of the optimal formulation (F4) and its individual components were analyzed using X-ray diffraction (XRD) patterns (Fig. 3). The crystallinity of APO was confirmed by distinct diffraction peaks at 2θ values of 13.125°, 22.611°, and 26.413° (Fig. 3a)^45^. The XRD pattern of CO, due to its inherent viscosity, exhibited a broad peak between 11.45° and 13.35° (Fig. 3b), with no other significant peaks^61^. In contrast, the XRD diffractograms of GE displayed moderately strong peaks at 21.32° and 23.55° (2θ) (Fig. 3c)^69^. The XRD profiles of CTOS revealed primary diffraction peaks at 2θ values of 9.2°, 12.5°, 19.0°, 20.9°, and 23.1° (Fig. 3d)^70^. The distinctive peaks of these components were also observed in the XRD pattern of the physical mixture (Fig. 3e), although the peaks associated with APO were diminished due to dilution. The diffractogram of the optimal formulation (F4) (Fig. 3g) closely resembled that of the plain formulation, with the disappearance of APO peaks indicating the loss of its crystalline structure as a result of encapsulation and the formation of amorphous NSLCs^71^.

##### Transmission electron microscopy (TEM)

**Fig. 4:**
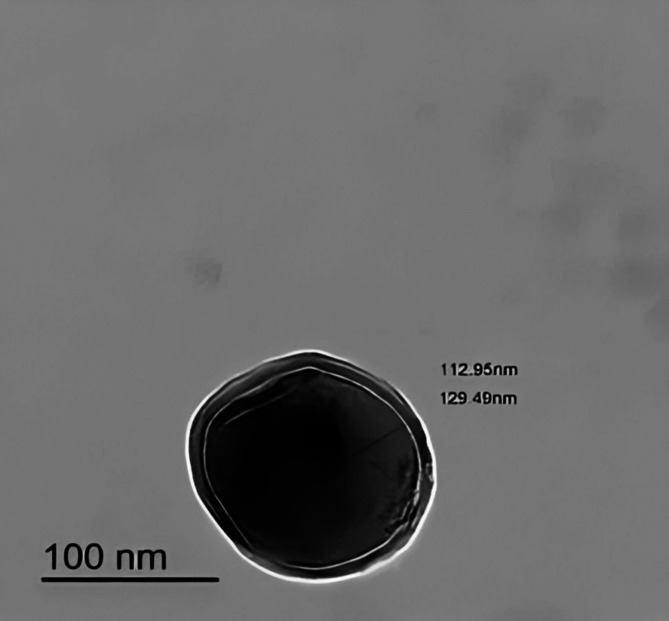
TEM images of APO-loaded NSLC optimal formula (F4).

Transmission Electron Microscopy (TEM) is an esteemed technique for probing the structural intricacies of nanoscale systems^45^. The structural configuration of nanomaterials plays a critical role in modulating their biodistribution and renal elimination kinetics^53^. The TEM photograph (Fig. 4) of the optimal formula (F4) reveals that the prepared NSLCs have spherical morphology with PS in the range of 100 nm (Fig. 1S**)**, confirming the results obtained by the Malvern Zeta sizer.

##### Scanning electron microscopy (SEM)

**Fig. 5:**
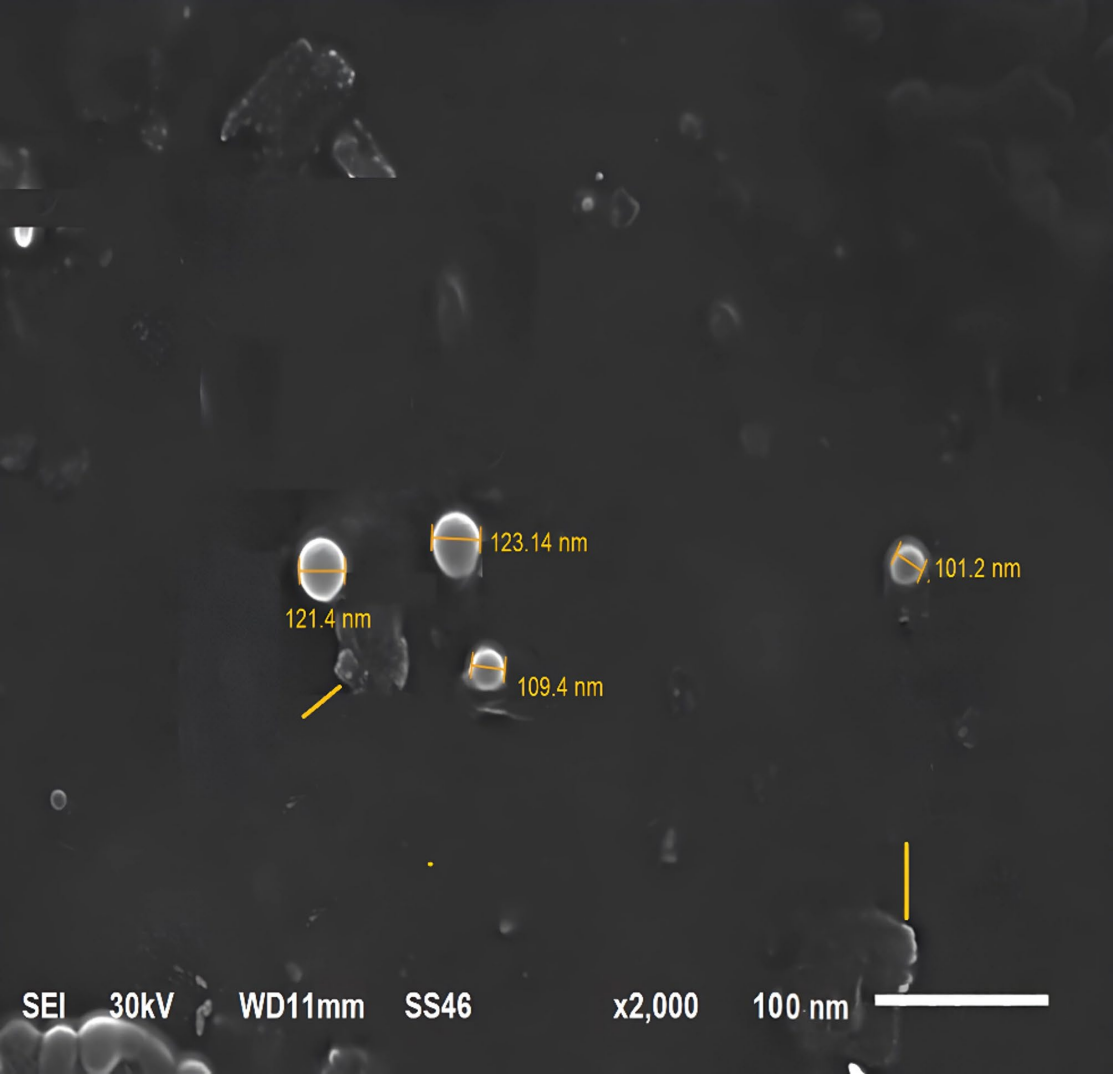
SEM images of the APO-loaded NSLCs optimal formula (F4).

The SEM images of the lyophilized APO-loaded NSLCs, depicted in Fig. 5, were utilized to explore their surface and size attributes, thereby validating the spherical structure of the NSLCs. Moreover, the particle size (PS) results from the SEM analysis (Fig. 5) were consistent with the PS measurements acquired using the Malvern Zetasizer, as presented in Table 2. The images revealed some clustering (yellow arrow), which could be associated with the shrinkage of the NSLCs during the drying process or the concentration of the dispersion medium^41^.

##### In vitro release study

**Fig. 6:**
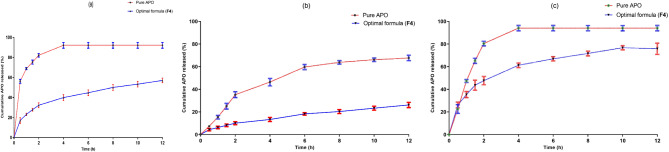
The in vitro release pattern of APO from aqueous solution in comparison with the optimal formula (F4) at three different pH media: **(a)** pH 1.2, **(b)** pH 6.8, and **(c)** pH 7.4. Each point represents the mean ± SD (*n* = 3).

The in vitro release patterns of APO from the optimal APO-loaded NSLCs (F4), as well as its diffusion from aqueous solution, were conducted utilizing three separate dissolution media, as represented in Fig. 6. The physicochemical properties of the lipid carrier and loaded drug dictated the release pattern of APO from both aqueous solution and APO-loaded NSLCs (**F4**)^72^. A burst release profile was evident in the first half hour from the beginning of the release for both pure APO and F4 at different pH values. This may be attributed to the diffusion of the free APO molecules adsorbed on the surface of the NSLCs^73^. Within 2 h, pure APO fully diffused from its aqueous solution into the release media at acidic (pH 1.2) and basic (pH 7.4) conditions, as illustrated in Fig. 6A **and C**. Such behavior of APO in both pH (1.2 & 7.4) is due to the amphoteric nature of APO, which might be attributed to the formation of hydrogen bonds between the release medium and the APO’s phenolic OH group^45^. However, at pH 6.8, free APO diffusion did not exceed 60% after 6 h and then remained steady (Fig. 6B). A probable justification may lie in the diminished ionization of APO at neutral pH, resulting in a subsequent reduction in its solubility. This aligns with findings reported by Anter et al.^8^. The in vitro release profiles of APO from the optimal formula (F4) at the three pH media displayed sustained release patterns over 12 h. The reason behind this sustainable release pattern was the slow erosion of the solid lipid (GE) matrix, which sustains the drug diffusion rate to the aqueous environment^74^. At pH 1,2 and 7.4, APO showed higher release profiles from the optimized NSLCs (F4) through the 12 h (45% and 60% respectively), compared to that at pH 6.8 (20%). The behavior observed may be elucidated by a likely decrease in the ionization degree of APO at neutral pH, as has been previously reported in the literature^45^.

###### Kinetic analysis of the drug release data

The drug release mechanisms and patterns observed in NSLCs are fundamentally influenced by their physicochemical properties. The R^2^ and AIC values detailed in Table 3 suggest that the diffusion of APO across different formulations aligns with the Higuchi-diffusion model, independent of the pH of the surrounding medium. Further analysis indicated that APO release was governed by a Fickian diffusion mechanism, as evidenced by n values below 0.5 at pH 1.2 and 7.4, with a non-Fickian mechanism being dominant solely at pH 6.8.

**Table 3:** **Kinetic** analysis of the drug release data of APO aqueous solution as well as the optimal formula (F4) (See Table 1 for **F4** composition).

Formula	pH	Zero-order		First order		Higuchi diffusion		Korsmeyer-Peppas			Main transport mechanism
		R^2^	AIC	R^2^	AIC	R^2^	AIC	R^2^	AIC	n	
Pure APO	1.2	0.40	99.9	0.52	65.40	0.65	88.98	0.81	57.03	0.17	Fickian
	6.8	0.84	76.52	0.90	63.11	0.94	56.91	0.91	58.91	0.76	non-Fickian
	7.4	0.55	96.26	0.61	54.92	0.77	82.99	0.73	75.34	0.48	Fickian
APO-NSLCs (F4)	1.2	0.79	77.2	0.86	45.69	0.93	53.98	0.92	24.61	0.41	Fickian
	6.8	0.89	48.76	0.90	39.28	0.93	18.43	0.90	7.72	0.65	non-Fickian
	7.4	0.72	86.84	0.84	74.99	0.90	67.94	0.91	45.86	0.38	Fickian

R^**2**^: coefficient of determination.

AIC: Akaike information criterion.

n: the characteristic diffusional exponent in Korsmeyer–Peppas kinetic equation.

##### Stability study

Table 4 summarizes the values of the investigated stability parameters: PS, PDI, ZP, and EE% of the optimal formula (F4) when kept over 6 months at two different conditions (ambient and refrigerated temperatures). The ANOVA results elucidated an insignificant variation in PS, PDI, ZP, and EE% throughout the storage period at refrigerated temperature. Contrarily, upon storage at ambient temperature, a significant elevation was recorded in PS, PDI, and a reduction in ZP and EE%. These findings indicate that the optimal formula (F4) remains highly stable when stored at refrigeration temperatures for six months, as reflected in its uniform size range and homogeneous distribution^45^. Previous research has also noted similar results regarding the storage stability of APO-loaded nanoparticles under the same temperature conditions^8^.

**Table 4:** Stability assessment parameters of the optimal formula (F4) following storage at different temperature conditions (See Table 1 for F4 composition).

Temp/humidity	Month	PS (nm)	PDI	ZP (mv)	EE%
**Refrigerated conditions** **4 ± 1 °C**	**0**	123.11 ± 2.21	0.14 ± 0.03	−28.12 ± 2.64	63.85 ± 1.25
	**1**	123.31 ± 1.32	0.14 ± 0.05	−27.96 ± 2.31	63.50 ± 1.32
	**2**	123.45 ± 1.75	0.14 ± 0.09	−27.85 ± 1.52	63.45 ± 1.21
	**3**	123.98 ± 1.52	0.15 ± 0.01	−27.64 ± 1.13	63.21 ± 1.13
	**4**	124.12 ± 0.14	0.15 ± 0.06	−27.36 ± 1.11	62.91 ± 1.85
	**5**	124.55 ± 0.19	0.15 ± 0.08	−27.21 ± 1.14	62.44 ± 1.55
	**6**	124.83 ± 0.55	0.16 ± 0.02	−27.11 ± 1.23	62.10 ± 2.32
**Ambient conditions** (25 °C ± 2 °C/60%RH ± 5% RH)	**0**	123.11 ± 2.21	0.14 ± 0.03	−28.12 ± 2.64	63.85 ± 1.25
	**1**	124.22 ± 1.11	0.17 ± 0.05	−27.81 ± 1.20	62.81 ± 1.21
	**2**	127.14 ± 1.52	0.18 ± 0.06	−27.11 ± 1.13	62.10 ± 1.51
	**3**	133.41 ± 1.98*	0.23 ± 0.00*	−25.92 ± 1.54	59.90 ± 1.22
	**4**	145.20 ± 2.1*	0.24 ± 0.01*	−23.41 ± 1.96*	56.41 ± 1.66*
	**5**	153.6 ± 2.22*	0.27 ± 0.02*	−21.63 ± 1.52*	53.21 ± 1.47*
	**6**	161.22 ± 2.5*	0.36 ± 0.05*	−21.12 ± 1.33*	51.52 ± 2.41*

Each value represents the mean ± SD (*n* = 3).

* Significant at *p* < 0.05 monthly vs. initial.

**PS**: particle size (nm), **PDI**: polydispersity index, **ZP**: zeta potential (mv), **E**E%: encapsulation efficiency (%).

##### In vivo assessment of the optimal APO-loaded NSLCs (F4) effect against FK506-induced AKI in rats

###### Effect of APO on FK506-induced change in body and kidney weights of rats

As depicted in Table 5, a significant (*p* < 0.05) body weight loss with an increase in kidney weight was observed in the positive control **(Group II)** when compared with the normal control (**Group I**). This loss in body weight may be attributed to either direct injury in renal tubules or increased catabolism^75,76^. Renal tubule injury results in the inability of the tubular cells to reabsorb water, leading to dehydration and loss of body weight, while the increased catabolism leads to acidosis, anorexia, and decreased food intake^75,77^. On the other hand, the increase in kidney weight after FK506 administration in the positive control group (**Group II**) was a result of inflammation and edema^78^. The results after concurrent treatment with pure APO (**Group III**) were nearly like those of the positive control (**Group II**) without any significant difference. However, concurrent treatment with APO-loaded NSLCs (**Group IV**) significantly reduced loss in body weight while preserving kidney weight compared to positive control and normal control groups (**Group II and I**,** respectively**). This result reflects the improvement in the APO oral bioavailability from the NSLC system in general and in renal tissue in particular. APO-loaded NSLCs (F4) provide a renal-targeted delivery for APO by their tunable size and surface charge (Figs. 1S and 2S). APO protects the renal tubules from the adverse effects of FK506 by reducing the activity of NADPH in the renal tissues, which in turn reduces the overproduction of ROS^3,79^. Furthermore, the antioxidant and anti-inflammatory properties of CO and CTOS play an important role in the reduction of the inflammatory cytokines such as TNF-α, IL-1, and IL-6^3,17,80^.

**Table 5:** Effect of APO on FK506-induced change in rats’ body weight and renal function.

Variable	Group I (Normal control)	Group II (Positive control)	Group III (Pure APO)	Group IV (APO-NSLCs)
Change in body weight (g)	10.5 ± 1.65	−17.4 ± 2.62*	−15.6 ± 1.31*	−5.2 ± 1.12*^# º^
Kidney weight (g)	0.75 ± 0.05	0.98 ± 0.02*	0.96 ± 0.03*	0.78 ± 0.02^# º^
Scr (mg/dL)	0.62 ± 0.05	0.89 ± 0.12*	0.83 ± 0.01*	0.65 ± 0.02^# º^
BUN (mg/dL)	28.1 ± 2.45	42.4 ± 5.62*	41.14 ± 1.51*	31.62 ± 2.98^# º^
Total protein (g/dL)	9.1 ± 2.2	4.8 ± 1.2*	4.9 ± 0.22*	8.5 ± 1.1^# º^
Cystatin C (ng/mL)	0.022 ± 0.0013	0.031 ± 0.0021*	0.029 ± 0.0020*	0.023 ± 0.0011^# º^
NGAL (pg/mL)	13.54 ± 3.41	22.63 ± 1.61*	21.51 ± 1.22*	15.12 ± 2.14^# º^
KIM-1 (pg/mL)	75.14 ± 5.12	124.32 ± 10.29*	119.25 ± 8.26*	79.25 ± 4.21^# º^

Scr: serum creatinine, BUN: blood urea nitrogen, NGAL: neutrophil gelatinase-associated lipocalin (NGAL), and KIM-1: kidney injury molecules.

*p < 0.05 significantly different from normal control.

^#^p < 0.05 significantly different from positive control.

^º^p < 0.05 significantly different from the Pure APO Group.

Using one-way ANOVA followed by Tukey -Kramer multiple comparisons post hoc test.

###### Effect of APO on FK506-induced changes in kidney function in rats

In the context of renal dysfunction, a decrease in glomerular filtration rate (GFR) leads to a compromised ability of the kidneys to filter creatinine (Cr), resulting in the accumulation of this nonprotein waste product. Additionally, there is an elevation in urinary protein levels, which may serve as a sensitive marker for tubular injury, diminished reabsorption of tubular proteins, or compromised filtration by the glomerular barrier, ultimately contributing to a decrease in serum protein levels^77^. The most obvious manifestation of FK506-induced nephrotoxicity is a rise in the serum creatinine concentration (Scr) as a result of a decline in the GFR. In the present study, FK506 induced a typical pattern of nephrotoxicity that was associated with a significant increase in serum Cr and BUN levels, with a reduction in serum total proteins (Table 5**)**. However, treatment with APO-loaded NSLCs maintains nearly normal levels of Scr, BUN, and total protein levels. The ameliorative effect of APO on the kidney markers may be attributed to the ROS scavengers and antioxidant molecules found in APO and CO that can partially reduce or eliminate the deleterious effects induced by FK506^3^. These results are in agreement with earlier reports^13,79,81^. Conversely, treatment with pure APO did not significantly reverse the changes induced by FK506 compared to the NSLCs loaded with APO (Table 5).

For the detection of initial renal injuries, it is crucial to utilize new biomarkers that demonstrate greater sensitivity and specificity than conventional ones, which are considered to have low sensitivity in identifying early renal damage^82^. Cystatin C (Cys. C) functions as a surrogate biomarker for glomerular filtration rate (GFR) and is not influenced by variables such as muscle mass, age, gender, or dietary habits. It is superior to serum creatinine (Scr) in that it can detect renal injury two days before any increase in blood urea nitrogen (BUN) and Scr levels^50^. Furthermore, serum levels of both neutrophil gelatinase-associated lipocalin (NGAL) and kidney injury molecule-1 (KIM-1) are sensitive and specific biomarkers that correlate with renal histopathological alterations during FK506-induced nephrotoxicity. The rise in these biomarkers is contingent upon the time elapsed and the dosage administered, as it corresponds to a progressive expression of genes. NGAL, a protein located in the renal proximal convoluted tubules, is also elevated following episodes of renal ischemia and injury. It can detect tubular damage in both in vitro and in vivo settings within a two-hour window post-renal ischemia^83^. KIM-1 is a type I transmembrane protein that cannot be detected in normal kidney tissue and urine. However, it is expressed at very high levels in the proximal tubules shortly after kidney injury^78^. KIM-1 serum levels are more sensitive than NGAL for the development of acute renal injury^83^.

Table 5 reveals that FK506 administration led to full prone nephrotoxicity reflected by increased tubular and glomerular damage biomarkers (Cys. C, NGAL, and KIM-1) in the positive control group (**Group II**), compared to the normal one (**Group I**). Concurrent administration of APO-loaded NSLCs (**F4**) with FK506 prevents the increase in renal tubular injury biomarkers (Cys. C, NGAL, and KIM-1). APO and CO prevent ROS production through restriction of renal-induced lipid peroxidation and, thus, stop renal damage^84^. Moreover, Statistical analysis of Table 5 results showed a significant difference between previous parameters in **Group III**, which was treated with pure APO aqueous suspension, and **Group IV**, which was treated with optimal NSLCs loaded with APO (**F4**). These results indicated that the incorporation of APO in the GE-based NSLCs improves renal delivery and accumulation of the APO by their tunable size and surface charge (Figs. 1S and 2S).

###### Histopathological analysis

**Fig. 7:**
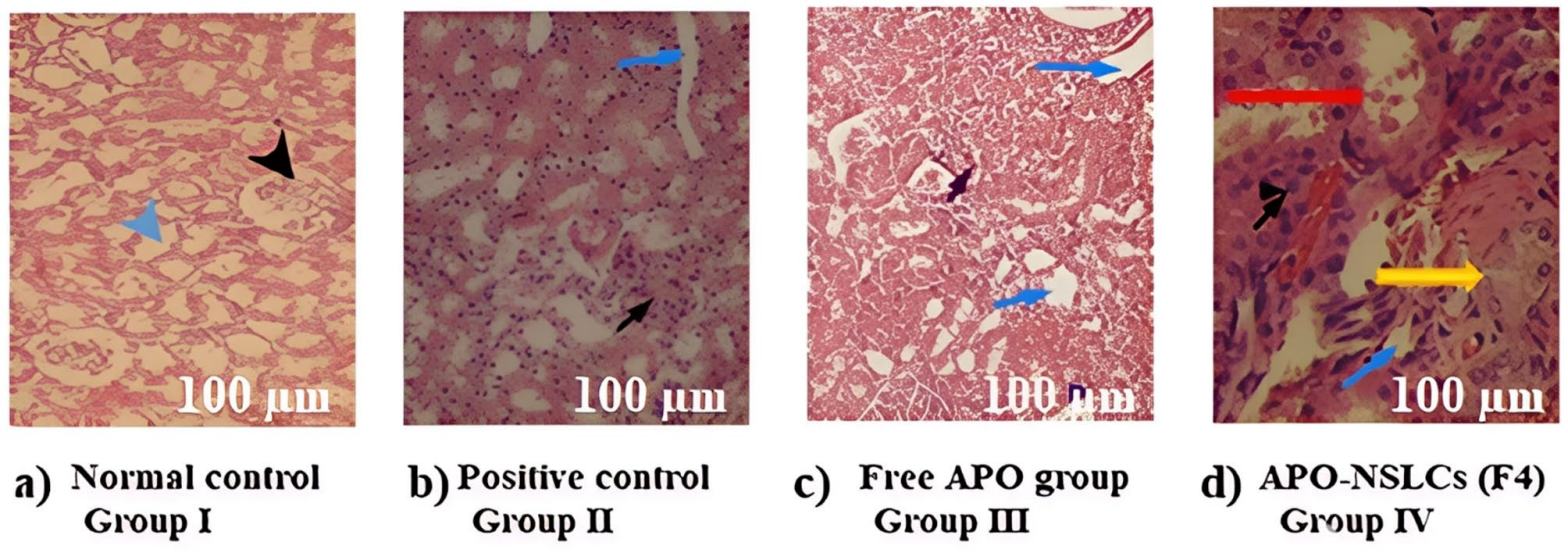
Effect of APO on FK506-induced histopathological changes in kidneys of different experimental groups: (**a**) Normal control group; (**b**) Positive control group; (**c**) Pure APO group, and (**d**) APO-NSLCs (F4). (*n* = 6 per group, Low magnification X:100, bar:100 μm).

Histopathological investigation of the kidney from rats of the normal control group (**Group I**) showed normal glomeruli (black arrowhead) and normal renal tubules with the normal lining of the renal tubular epithelium (blue arrowhead) (Fig. 7a). By contrast, nephropathic changes were observed in the positive control group (**Group II**) upon treatment of the rats with FK506 for seven days. The glomerulus showed an enlargement of renal glomeruli and epithelial cells in the cortical part of the kidney (black arrowhead). The tubules showed signs of toxicity in the form of dilated, irregular tubules with attenuated lining, loss of brush border, and a few apoptotic cells (blue arrowhead) (Fig. 7b). Mild interstitial inflammation, tubular vacuolization, atrophy in the cortex and outer medulla, with moderate interstitial edema, and tubular necrosis were observed in the group treated with pure APO (**Group III**) (Fig. 7c). **However**,** Group IV**, which was treated with the optimal NSLCs loaded with APO **(**Fig. 7D**)**, showed an intact glomerulus (black arrow) and normal lining of the renal tubular epithelium (blue head arrow). Some tubules showed a sign of injury in the form of cellular blebs or apoptotic cells (red arrow), with areas showing vesicular nuclei with prominent nucleoli (a sign of regeneration – yellow arrow). The overall findings of the histopathological examination depict that concurrent treatment with unformulated APO (**Group III**) did not affect the histopathological damages induced by FK506, while the optimal NSLC loaded with APO was associated with the normalization of renal cell damage, as the treated rats showed nearly normal cellular features. The toxic effects of FK506 are thought to stimulate renal oxidative stress since it facilitates ROS formation. The renal protection of APO and CO is thought to be stimulated by their antioxidant properties. Both reduce ROS production and lead to an amelioration of FK506-induced nephrotoxicity in rats. APO ameliorated tubular necrosis and glomerular alterations and induced apoptotic cell death^85^. In addition, CO’s main constituent (eugenol) may reinforce the body’s endogenous antioxidants that are diminished due to FK506 administration, thereby preventing the acute tubular necrosis^86^. Fruitfully, these findings, when considered alongside our previously published results, collectively reinforce the therapeutic relevance of apocynin (APO) as a phytopharmaceutical agent^35^. Complementary data underscores APO’s efficacy in attenuating oxidative stress and its translational potential in the treatment of both cystitis and nephritis^35^.

## Conclusion

This pioneering investigation highlights the therapeutic potential of apocynin (APO)-loaded nanostructured lipid carriers (NSLCs) as a phytopharmaceutical strategy for mitigating FK506-induced nephrotoxicity. The optimized NSLC formulation (**F4**) achieved high encapsulation efficiency (**EE**%: 63.85 ± 1.98) and demonstrated favorable nanoscale physicochemical characteristics, including a **PS** of 123 ± 2.21 nm, a negative **ZP** of 28 ± 1.98 mV, **PDI** of 0.17 ± 0.09, and spherical morphology, ensuring a homogeneous distribution and sustained release profile. Furthermore, F4 remained physically stable under refrigerated conditions for six months, supporting its pharmaceutical robustness and storage suitability. Concurrent oral administration of F4 with FK506 significantly ameliorated nephrotoxicity, as evidenced by improved serum biomarkers and renal histopathology. The therapeutic efficacy of this system stems from the physicochemical properties of the NSLCs, which enhance APO’s renal targeting and retention. The coordinated action of the phytopharmaceutical triad (APO, CO, and CTOS) was crucial to modulating oxidative and inflammatory responses. APO notably downregulated NADPH oxidase activity, resulting in reduced ROS generation, while polyphenols present in APO and CO attenuated the formation of highly toxic hydroxyl radicals. Taken together, the results position APO-loaded NSLCs as a promising phytotherapeutic modality for mitigating immunosuppressant-induced nephrotoxicity. Considering the continued clinical reliance on FK506 and similar agents in transplant immunotherapy, long-term in vivo investigations are warranted to validate the chronic safety and efficacy of this advanced nanocarrier system.

## Supplementary Information

Below is the link to the electronic supplementary material.

Supplementary Material 1

## Data Availability

The datasets generated during and/or analyzed during the current study are available from the corresponding author on reasonable request.
