# Supplementary material for: Innovative nanostructured lipid-particles of apocynin and clove oil tagged with Chitin oligosaccharide for amelioration of tacrolimus-induced nephrotoxicity
Source: Sci Rep. 2025 Aug 8;15:29011. doi: 10.1038/s41598-025-13978-1 (PMC12334713; doi:10.1038/s41598-025-13978-1)
Supplement: Supplementary file 1 — Supplementary Material 1 [file 41598_2025_13978_MOESM1_ESM.docx]

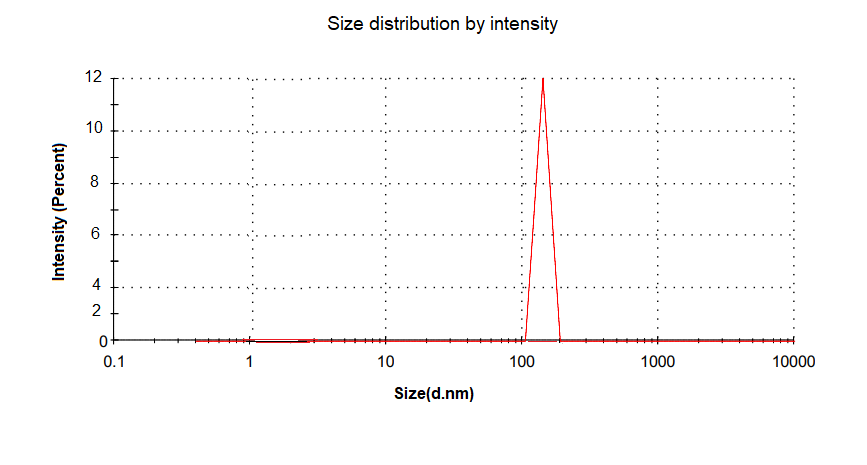


**Fig. 1S**. Particle size distribution by intensity of the optimum formula (F4)


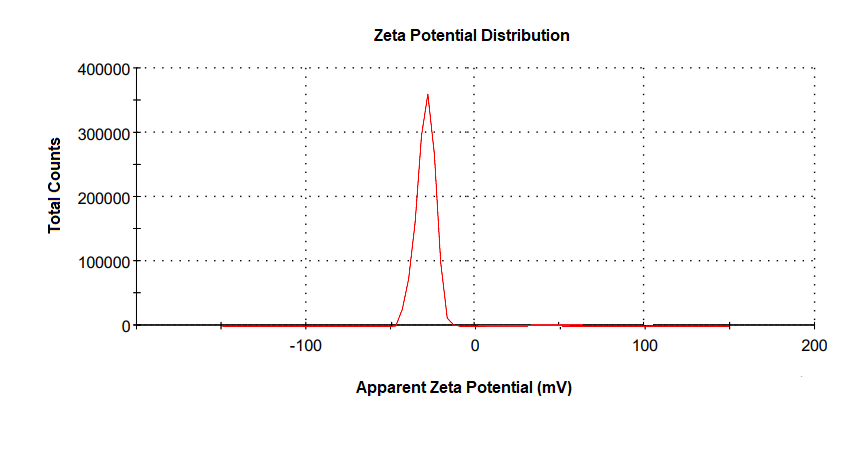


**Fig. 2S**. Zeta potential distribution of the optimum formula (F4)
